# Supplementary material for: Quantitative characterization of extracellular vesicle uptake and content delivery within mammalian cells
Source: Nat Commun. 2021 Mar 25;12:1864. doi: 10.1038/s41467-021-22126-y (PMC7994380; doi:10.1038/s41467-021-22126-y)
Supplement: Supplementary file 1 — Supplementary Information [file 41467_2021_22126_MOESM1_ESM.pdf]

Supplementary Figure 1

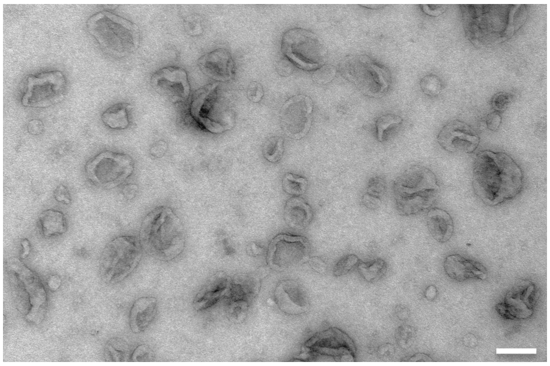

Supplementary Figure 1: EM observation of Isolated Evs.
EVs were isolated through sequential centrifugation and processed for Electron Micocroscopy. Bar, 100 nm.

Supplementary Figure 2

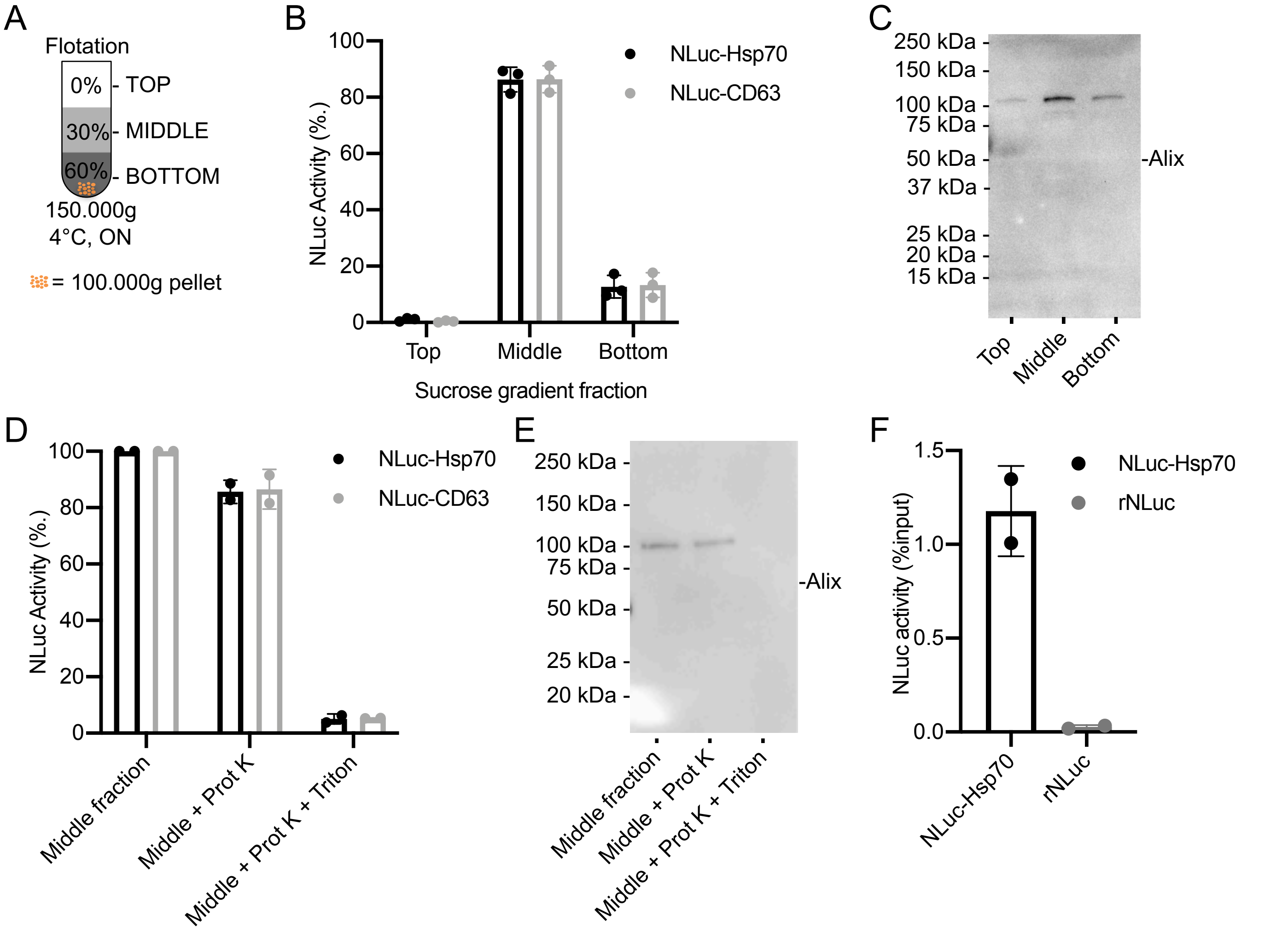

Supplementary Figure 2: NLuc-Hsp70 and NLuc-CD63 EV characterization using sucrose gradient

A: Scheme of the EV isolation through floatation assay.

B: NLuc activity distribution in sucrose fractions after isolation of EVs emanating from NLuc-Hsp70 (black) and NLuc-CD63 (grey) HeLa cells. NLuc activity was measured in each fraction of the sucrose gradient. Sum of the NLuc activity of the 3 fractions was set to 100%. Each dot is an independent replicate and represents the mean of 2 technical replicates, n=3. Error bars represent standard deviations.

C: Alix distribution in sucrose fractions. Immunoblot of the 3 collected fractions from sucrose gradient were tested for Alix. Equal volume was loaded for each fraction. This blot is representative of 2 independent experiments.

D: Protease-protection assay on the 30% sucrose-EVs (middle fraction) emanating from NLuc-Hsp70 (black) or NLuc-CD63 (grey) HeLa cells. Middle fraction-EV were treated or not with proteinase K, with or without detergent. NLuc activity from nontreated sample (control) was set to 100%. Each dot is an independent replicate and represents the mean of 2 technical replicates, n=2. Error bars represent standard deviations.

E: Protease protection assay on Alix, on middle-fraction EVs emanating from NLuc-Hsp70 or HeLa cells. Presence of Alix was tested by Immunoblot. This blot is representative of 2 independent experiments.

F: Uptake of middle fraction-EVs from NLuc-Hsp70 cells. Isolated floating NLuc-Hsp70 EVs (black) or recombinant soluble NLuc (rNLuc, grey) were incubated with unlabeled HeLa WT acceptor cells for 1h. NLuc activity associated with acceptor cells was measured after incubation. NLuc activity for EV or rNLuc initial input were set to 100% uptake. Each dot is an independent replicate and represents the mean of 2 technical replicates, n=2. Error bars represent standard deviations.

Supplementary Figure 3

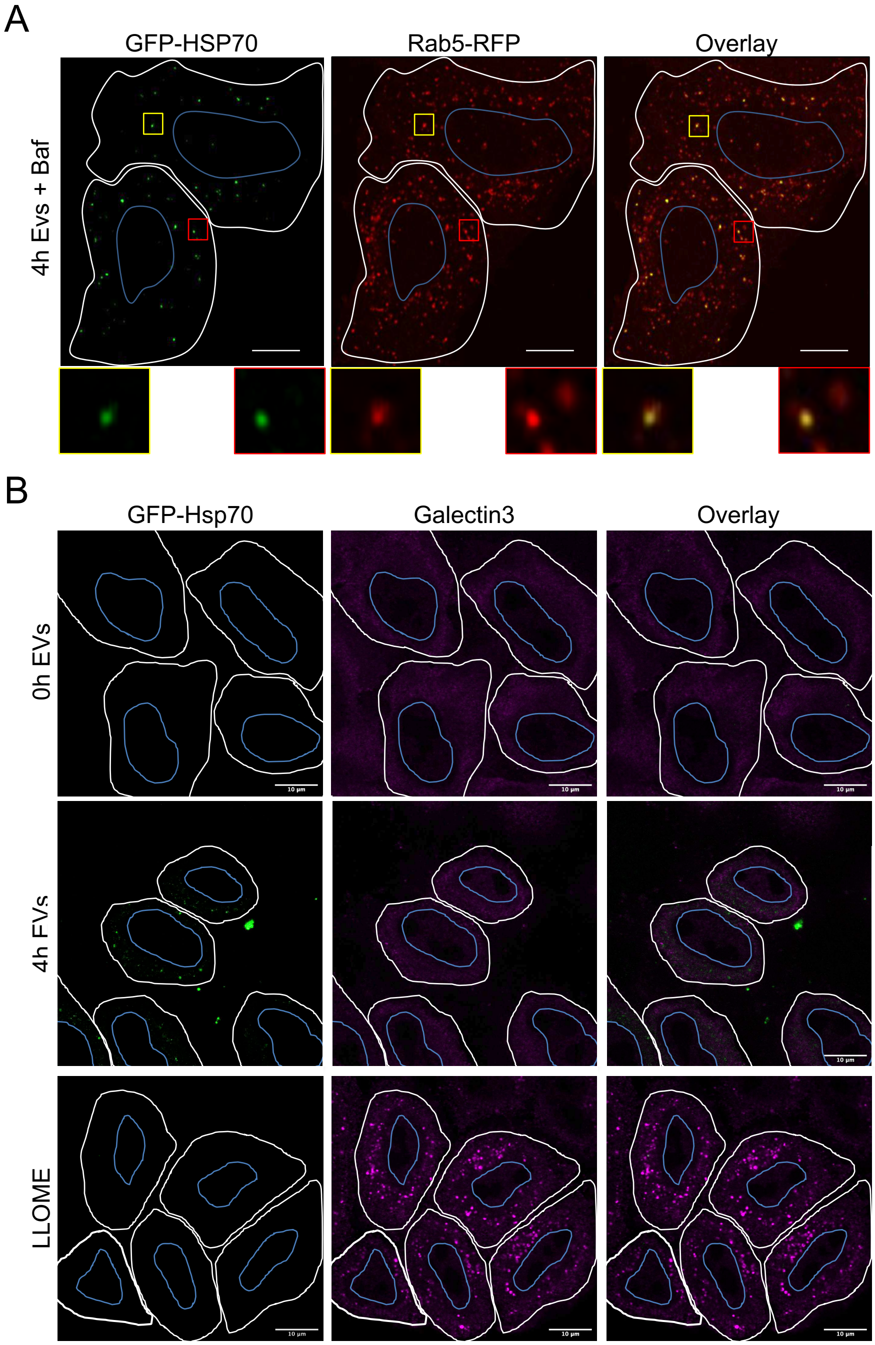

Supplementary Figure 3: Endo/lysosomal acidification is required for EV content delivery
A: BafilomycinA1 treatment confined EV content in endosomal compartments. GFP-Hsp70 EVs were loaded for 4 hours on unlabeled HeLa WT acceptor cells, treated with BafilomycinA1. Cell were immuno-labeled against Rab5. Micrographs representative of 2 independent experiments. Barre,10 µm

B: Endosomal integrity after incubation with GFP-Hsp70 EV. Isolated GFP-Hsp70 EVs, were loaded for 4 hours on unlabeled HeLa WT acceptor cells, which were fixed and immuno-labeled against Galectin3. Cells were treated 30min with 500mM LLOME as positive control for endosomal damage. Micrographs representative of 3 independent experiments. Barre,10 µm

Supplementary Figure 4

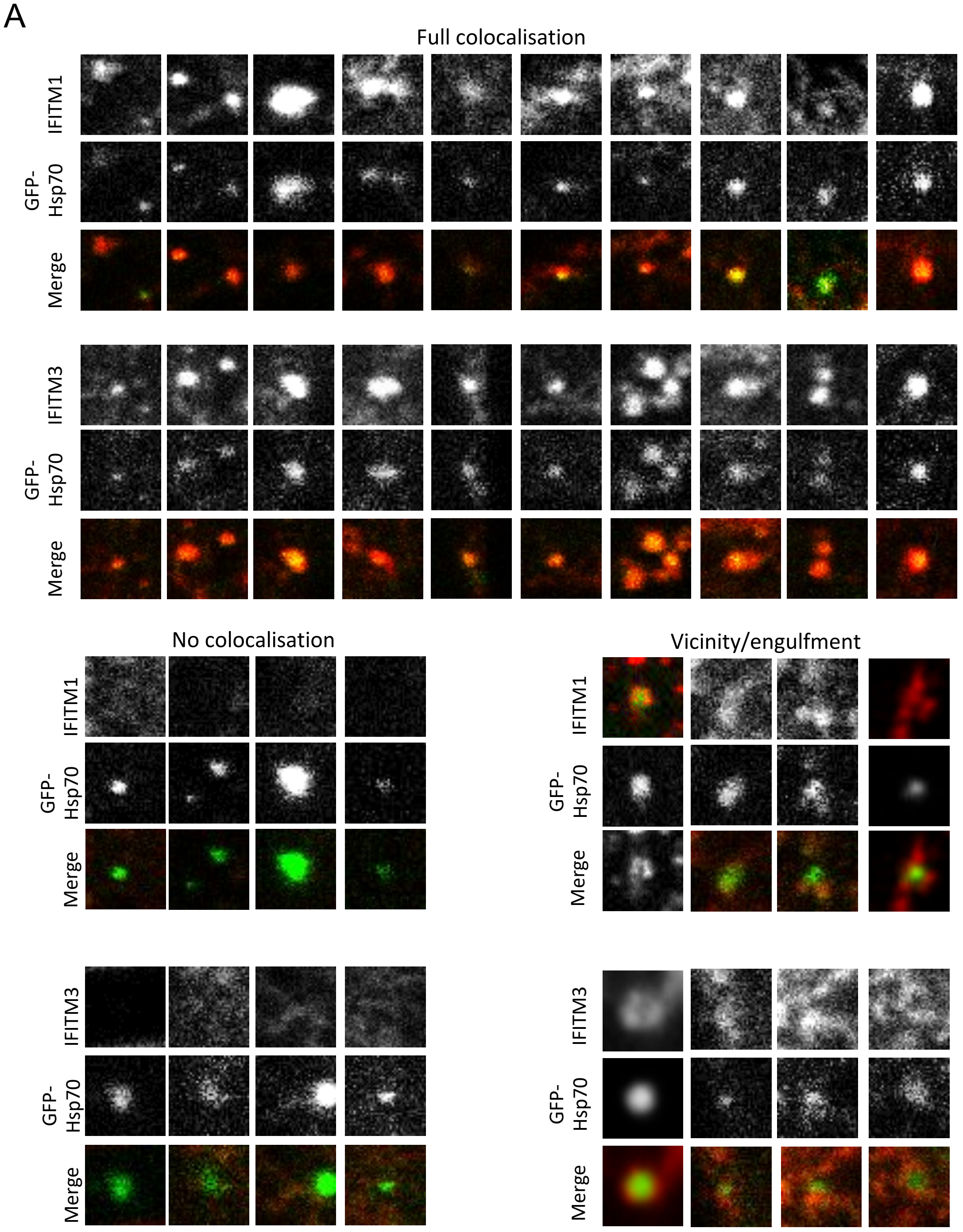

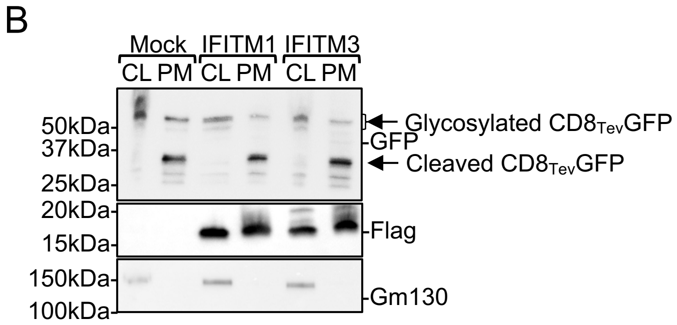

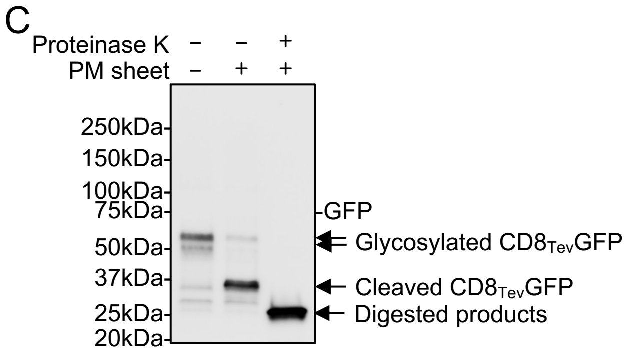

Supplementary Figure 4: IFITM proteins inhibit EV content delivery

A: Micrographs showing GFP positive ROIs where IFITM fluorescent signals fully colocalize, or where GFP foci are engulfed in IFITM positive structures. In few cases (<8-16 %, for IFITM3 and 1, respectively) GFP foci do not colocalize with IFITM positive objects. Micrographs representative of 2 independent experiments.

B: Immunoblots of cell lysate (CL) or purified plasma membranes (PM) from HEK cells expressing CD8TevGFP alone (mock), or with IFITM1-Flag or IFITM3-Flag proteins. Samples were tested for Flag, GFP, and Gm130 proteins. This blot is representative of 2 independent experiments.

C: Immunoblots of CL of PM from HEK cells expressing CD8TevGFP treated or not with proteinase K, which generates GFP-positive digested products observed in Figure 6.F(lower bands). This blot is representative of 2 independent experiments.

Supplementary Table 1

Primer name	Primer sequence
F-CD63-AgeI-NLuc	5’-ATTACTACCGGTATGGTCTTCACACTCGAAGATTTC-3’
R-CD63-XhoI-NLuc	5’-ATTACT**CTCGAG**CGCCAGAATGCGTTCGCACAG-3’

Supplementary Table 1: Primer sequence for NLuc cloning
Primers were designed to amplify NLuc sequence with restriction sites that will allow ligation within acceptor Hsp70 or CD63 plasmids.
